# Mechanistic Insights into Alzheimer’s Disease Unveiled through the Investigation of Disturbances in Central Metabolites and Metabolic Pathways

**DOI:** 10.3390/biomedicines9030298

**Published:** 2021-03-14

**Authors:** Raúl González-Domínguez, Álvaro González-Domínguez, Ana Sayago, Juan Diego González-Sanz, Alfonso María Lechuga-Sancho, Ángeles Fernández-Recamales

**Affiliations:** 1 AgriFood Laboratory, Faculty of Experimental Sciences, University of Huelva, 21007 Huelva, Spain; ana.sayago@dqcm.uhu.es (A.S.); recamale@dqcm.uhu.es (Á.F.-R.); 2 International Campus of Excellence CeiA3, University of Huelva, 21007 Huelva, Spain; 3 Inflammation, Nutrition, Metabolism and Oxidative Stress Study Group (INMOX), Biomedical Research and Innovation Institute of Cádiz (INiBICA), Research Unit, Puerta del Mar University Hospital, 11009 Cádiz, Spain; alvaro.gonzalez@inibica.es (Á.G.-D.); alfonso.lechuga@uca.es (A.M.L.-S.); 4 Department of Nursing, COIDESO Research Center, University of Huelva, 21007 Huelva, Spain; juan.gonzalez@denf.uhu.es; 5 Pediatric Endocrinology, Department of Pediatrics, Puerta del Mar University Hospital, 11009 Cádiz, Spain; 6 Area of Pediatrics, Department of Child and Mother Health and Radiology, Medical School, University of Cádiz, 11002 Cádiz, Spain

**Keywords:** central metabolites, Alzheimer’s disease, mass spectrometry, central metabolic pathways

## Abstract

Hydrophilic metabolites are closely involved in multiple primary metabolic pathways and, consequently, play an essential role in the onset and progression of multifactorial human disorders, such as Alzheimer’s disease. This review article provides a comprehensive revision of the literature published on the use of mass spectrometry-based metabolomics platforms for approaching the central metabolome in Alzheimer’s disease research, including direct mass spectrometry, gas chromatography-mass spectrometry, hydrophilic interaction liquid chromatography-mass spectrometry, and capillary electrophoresis-mass spectrometry. Overall, mounting evidence points to profound disturbances that affect a multitude of central metabolic pathways, such as the energy-related metabolism, the urea cycle, the homeostasis of amino acids, fatty acids and nucleotides, neurotransmission, and others.

## 1. Alzheimer’s Disease and Metabolomics: The Challenge of Hydrophilic Metabolites

Alzheimer’s disease (AD) is nowadays a major health problem due to the dramatic population aging worldwide. As this neurodegenerative disorder presents great variability of complex clinical symptoms and a long pre-symptomatic period, the underlying etiological factors remain a tangled meshwork to be unraveled. In this respect, mounting evidence points to a multifactorial and systemic crosstalk of heterogeneous pathological mechanisms, encompassing the well-known proteopathies associated with the deposition of β-amyloid plaques and the hyper-phosphorylation of tau protein, but also other cellular perturbations related to oxidative stress and inflammation, energy-related disturbances, altered neurotransmission, and metal homeostasis, among others [1,2,3]. Considering this multifaceted nature, numerous authors have extensively explored the potential of metabolomics for holistically elucidating the characteristic molecular alterations behind the onset and progression of AD [4,5]. In this vein, nuclear magnetic resonance (NMR) and reversed-phase liquid chromatography coupled to mass spectrometry (RPLC-MS) are currently the gold standard techniques in metabolomics, and most of the literature published on AD research is based on their application to brain, cerebrospinal fluid (CSF), blood, and other biological matrices. However, it is recognized that the low sensitivity and low spectral resolution of NMR considerably reduce its coverage, thus impeding comprehensive metabolomics analysis [6]. On the other hand, although great efforts have been made to develop large-scale RPLC-MS metabolomics approaches [7], this analytical platform is usually limited to the determination of low and medium polarity metabolites, such as lipids (i.e., lipidomics) [8], aromatic amino acids and their microbiota derivatives [9], some nutrients and food-related metabolites (i.e., nutrimetabolomics) [10,11], and a few other metabolite classes. Accordingly, the efficient profiling of hydrophilic metabolites is still a methodological challenge. Hydrophilic metabolites comprise multiple chemical classes, including sugars, most amino acids and derivatives, biogenic amines, organic acids, and many others, which are in turn involved in a multitude of central metabolic pathways, such as the energy-related metabolism (e.g., glycolysis, tricarboxylic (TCA) cycle), the urea cycle, the one-carbon metabolism, and others. Therefore, orthogonal analytical tools are crucial to approach this essential piece of the human metabolome puzzle, namely direct mass spectrometry (DMS), gas chromatography-mass spectrometry (GC-MS), hydrophilic interaction liquid chromatography-mass spectrometry (HILIC-MS), and capillary electrophoresis-mass spectrometry (CE-MS). 

DMS fingerprinting, based on the direct introduction of the sample extracts into the mass spectrometer, shows great utility for high-throughput and comprehensive metabolomics analysis thanks to the lack of a chromatographic or electrophoretic separation prior to MS detection, which inherently bias the method coverage [12]. However, this screening tool suffers from considerable ion suppression and the impossibility of resolving isomeric metabolites, which make the use of complementary approaches mandatory. Among MS-based hyphenated methods, GC-MS has been widely employed in metabolomics because of its reproducibility, chromatographic resolution, sensitivity, and selectivity [13]. This technique usually requires the application of a derivatization process before the analysis for increasing the volatility and thermal stability of metabolites, thus enabling the profiling of numerous low molecular weight central metabolites, such as amino acids, sugars, organic and fatty acids, amines, and many other primary metabolites. To avoid this derivatization step, which may represent an important source of variability and bias, the use of HILIC-MS is significantly increasing over the last years to analyze hydrophilic metabolites [14]. Complementarily, the coupling CE-MS provides orthogonal separation performance to LC and GC approaches for the analysis of highly polar and ionic metabolites [15]. Nonetheless, HILIC and CE present serious reproducibility-related limitations (e.g., drifts in retention/migration times along sequence runs) and reduced sensitivity compared with the most robust RPLC-MS and GC-MS platforms, which consequently hinder their application in large-scale metabolomics. Although much less employed in metabolomics, other RPLC-based alternatives for approaching polar metabolites include the use of ion-pair agents or derivatization to reduce the polarity of hydrophilic metabolites. However, ion pairing may provoke ion suppression during MS analysis, contamination of the MS ion source, and column instability, whereas common derivatization protocols (e.g., dansylation) are time-consuming and considerably reduce the method coverage toward specific metabolites classes that are sensitive to the derivatizing reagent. As a complementary approach, the use of imaging mass spectrometry is gaining great importance for in situ metabolomics analysis to map the molecular mechanisms underlying neurodegenerative disorders [16]. These techniques can complement traditional MS platforms relying on the investigation of postmortem and peripheral biofluid samples, thus facilitating the association between metabolomics and histological data. However, the application of these tools in AD research is still scarce and mainly limited to the lipidome [16]. Therefore, considering the inherent limitations of each one of all the platforms usually employed in metabolomics, more and more authors have emphasized the benefit of combining complementary techniques to obtain comprehensive metabolomics coverage [17].

The next sections of this review article provide an overview of the literature published over the last years aimed to explore the AD-characteristic disturbances in central metabolites and associated metabolic pathways through the application of the MS-based metabolomics platforms that are described above, as summarized in Table 1. The literature search was conducted in three online databases (Scopus, Web of Science, PubMed), using the search terms “Alzheimer”, “metabolomics”, “mass spectrometry”, “gas chromatography”, “hydrophilic interaction liquid chromatography”, ”capillary electrophoresis”, and “direct infusion”. Studies not focused on the hydrophilic metabolome were discarded.

## 2. Alzheimer’s Disease and DMS-Based Metabolomics

DMS analysis has been repeatedly applied in various AD metabolomics studies as a first-pass screening tool for simultaneously measuring a wide range of metabolite classes, including hydrophilic metabolites and lipids, in a high-throughput manner [68]. Two-step extraction of serum samples from AD patients and subsequent DMS fingerprinting revealed significant perturbations in the circulating levels of energy-related metabolites (e.g., glucose, fatty acids, and carnitine involved in β-oxidation) and neurotransmitters (e.g., dopamine) [18]. Additionally, it also found an abnormal phospholipid homeostasis reflected in reduced levels of species containing polyunsaturated fatty acids, increased levels of phospholipids composed of saturated fatty acids, and increased content of breakdown products (e.g., choline). Interestingly, this was then corroborated in other DMS-based works focused on the serum AD-related lipidome [19,20] and phospholipidome [21]. Although the electrospray (ESI) source is the most common ionization technique employed in DMS analysis, the atmospheric pressure photoionization (APPI) source has demonstrated complementary performance and metabolomics coverage [22]. In this work, the authors reported an accumulation of diacylglycerols, free fatty acids, and ceramides in AD serum, which is in line with the upregulated degradation of membrane lipids that was hypothesized in previous DMS studies, as well as other disturbances related to the monoaminergic neurotransmission and the urea cycle. These DMS-based high-throughput metabolomics platforms have also been employed to investigate the AD-like pathology in the APP × PS1 transgenic mouse model by considering multiple biological matrices, namely serum [23], urine [24], brain [25], and other peripheral organs [26]. The analysis of serum samples evidenced comparable metabolic disturbances to those reported in human studies, thus corroborating the utility of this transgenic line to model AD [23]. A similar strategy was applied to investigate the effect of interleukin-4 depletion (i.e., IL4-KO) on AD pathology by using APP × PS1×IL4-KO mice [27]. The results showed reduced serum content of various amino acids and metabolites implicated in the urea cycle, and the accumulation of eicosanoids in the IL4-KO model, which supported the close link between AD and inflammatory processes. Metabolomics fingerprinting of brain tissue enabled the in situ and region-specific investigation of the neuropathological processes underlying this neurodegenerative disorder [25]. Hippocampus and cortex were characterized by profound alterations in the levels of numerous lipids (e.g., phospholipids, fatty acids, acyl-carnitines, steroids) and hydrophilic metabolites (e.g., amino acids and derivatives), whereas other brain areas such as the cerebellum and olfactory bulbs were less affected. Complementarily, other peripheral organs, including the liver, kidneys, spleen, and thymus, were also studied to evaluate the systemic manifestations of the molecular mechanisms behind the AD pathology [26]. In this line, Lin et al. applied DMS metabolomics to characterize the metabolic perturbations in hippocampus [28] and cerebellum [29] of the CRND8 transgenic mouse. Interestingly, the most important findings were related to an altered metabolism of arachidonic acid and eicosanoids, amino acids, nucleotides, and other metabolite classes, which were in great agreement with the studies performed on the APP × PS1 model.

## 3. Alzheimer’s Disease and GC-MS Based Metabolomics

Metabolomics based on GC-MS has been successfully applied to various biological samples for investigating the impact of AD on primary metabolic pathways. Widespread disturbances related to the glucose metabolism, the urea cycle, and amino acid homeostasis were detected in seven brain regions from AD patients (hippocampus, entorhinal cortex, middle-temporal gyrus, cingulate gyrus, sensory cortex, motor cortex, cerebellum), including some regions traditionally considered not to be affected [30]. Similar alterations were observed in the hippocampus of the SAMP8 mouse along a 10-month follow-up, comprising energy-related metabolites, amino acids, lipids, and some others [31]. In this regard, Han et al. have demonstrated that these hippocampal metabolic perturbations may be sharpened by chronic unpredictable mild stress in APP × PS1 mice, particularly in relation to the metabolism of amino acids, ketone bodies, and sphingolipids [32]. Furthermore, comparative analysis of brain and plasma from TASTPM mice revealed the occurrence of similar disturbances in both biological matrices, thus reinforcing the utility of peripheral blood to mirror the neuropathological changes underlying AD [33]. In another study, these systemic manifestations on the AD-related metabolome were reflected in altered levels of 23 metabolites in serum from AD patients, indicating impaired neurotransmission, energy metabolism (e.g., TCA cycle), urea cycle, and some others [34]. Finally, other samples analyzed by GC-MS in AD metabolomics research included exhaled breath [35] and urine [36], with the aim of investigating the involvement of volatile compounds and odorants in its etiology.

The combination of GC-MS with RPLC-MS is nowadays one of the most common strategies to achieve comprehensive metabolomics analysis. This multiplatform was applied to serum samples from a prospective study among AD, mild cognitive impairment (MCI), and healthy subjects, uncovering profound lipidomics changes in AD patients at baseline and increased 2,4-dihydroxybutanoic acid along disease progression [37]. Wang et al. found that plasma metabolites measured through GC+RPLC-MS might serve to differentiate AD and amnesic MCI patients from control subjects [38]. The metabolic signatures associated with AD and MCI shared many metabolites participating in the metabolism of fatty acids, amino acids, nucleic acids, and one-carbon metabolism, thus suggesting the occurrence of common pathogenic mechanisms in both dementia disorders. In a more recent study, plasma metabolomics revealed that AD among the Down syndrome population is characterized by a shift in energy metabolism toward the upregulation of the anaerobic respiration [39]. In this line, it has been reported that mitochondrial stress and altered energy metabolism are major hippocampal disturbances occurring in three transgenic models of AD [40]. Metabolomics analysis of CSF has also been proposed to identify potential biomarkers for an improved diagnostic performance of AD. Czech et al. described that the combination of cortisol, cysteine, and uridine levels together with other amino acids yields predictive models with sensitivity and specificity above 80% [41]. Another study detected two CSF metabolic features with higher discrimination performance than that provided by traditional amyloid and tau biomarkers, but further confirmatory studies are needed [42]. To complement the DMS screening analyses that have been described in the previous section of this review article [18,19,20,21,22,23,24,25,26,27], González-Domínguez et al. conducted a comprehensive metabolomics characterization of the APP × PS1 model by applying combined GC-MS and RPLC-MS analysis to serum samples [43], various brain regions [44], metabolically active organs (i.e., liver, kidney) [45], and organs involved in the immune function (i.e., spleen, thymus) [46]. These studies validated most of the findings unveiled by DMS-based metabolomics fingerprinting with regard to the altered homeostasis of lipids (e.g., phospholipids, sphingolipids, cholesterol, fatty acids and acyl-carnitines), amino acids, nucleic acids, energy-related metabolism, and many others, both at the central and the systemic levels.

## 4. Alzheimer’s Disease and HILIC-MS-Based Metabolomics

Although its use is not as widespread as other metabolomics platforms such as RPLC-MS and GC-MS, various published works have explored the potential of HILIC-MS to investigate the AD-related polar metabolome. Two preliminary studies demonstrated that the statistical modeling of HILIC-MS metabolomics data may provide satisfactory subject classification by using both brain [47] and plasma [48] samples, but the metabolites responsible for this discrimination were not identified. In another prospective study, the analysis of plasma samples from MCI patients, MCI patients who developed AD upon the follow-up and healthy controls revealed disturbances in 22 metabolic pathways, some of them traditionally associated with the pathogenesis of AD (e.g., metabolism of cholesterol, glucose, and amino acids), but also in relation to the polyamine and the L-arginine metabolism [49]. Recently, urinary metabolomics also highlighted the pivotal involvement of aromatic amino acids in the CRND8 mouse model, encompassing alterations in the tryptophan metabolism (e.g., upregulation of the serotonin pathway, downregulation of the kynurenine pathway), deficient aromatic L-amino acid decarboxylase activity (e.g., accumulation of N-acetylvanilalanine and 3-methoxytyrosine), and changes in microbiota-related and glycine conjugation processes [50].

Orthogonal RPLC and HILIC separations have also been used in combination to maximize the metabolomics coverage, as previously described for the RPLC+GC multiplatform. Trushina et al. accomplished a comprehensive investigation of the metabolic mechanisms behind the onset of AD and MCI by analyzing plasma and CSF samples, which revealed alterations in multiple pathways associated with energy metabolism, mitochondrial function, neurotransmission, amino acid and lipid metabolism, and many others [51]. A similar analytical approach was employed to predict the progression of AD along four diagnostic groups, namely healthy control subjects, stable MCI patients, MCI subjects who developed AD after a 2-year follow-up, and AD patients, which indicated significant alterations in the CSF levels of some amino acids and taurine-related metabolites [52]. Comparable findings have been obtained from brain metabolomics profiling, thus suggesting that modulating the metabolism of amino acids could be a possible therapeutic approach against AD [53]. Additionally, in the brain, Paglia et al. found profound cortical perturbations in the metabolism of glycerophospholipids and six central metabolic pathways, of which a significant impairment of the mitochondrial aspartate metabolism was noteworthy [54]. Conversely, other authors were surprisingly not able to differentiate AD patients and healthy controls by using combined RPLC+HILIC-MS plasma metabolomics, but a clear discrimination was achieved between MCI and control subjects [55].

## 5. Alzheimer’s Disease and CE-MS Based Metabolomics

The application of CE-MS based metabolomics in AD research has only been reported in five studies published up to date. Ibáñez et al. identified a panel of CSF polar metabolites that was able to differentiate among subjects with different cognitive status, including patients with subjective cognitive impairment (SCI), MCI patients that remained stable within 2 years, MCI patients that progressed to AD after this follow-up period, and AD patients [56]. In another cohort, the analysis of serum samples evidenced similar alterations in metabolites related to oxidative stress, deficiencies in energy metabolism, and vascular risk factors [57]. Furthermore, the authors also found increased serum levels of proline betaine in AD patients, which is a marker of citrus consumption that has been recently validated in a prospective study on cognitive decline [69]. In this line, the analysis of brain tissue demonstrated that AD pathogenesis could be associated with dysregulated transmethylation and polyamine metabolism, abnormalities in neurotransmission, and impaired urea cycle and glutathione synthesis [58]. The two other studies published on CE-based metabolomics focused on the comparison of various neurodegenerative disorders. Tsuruoka et al. identified six serum metabolites and two saliva metabolites that were significantly altered in dementia patients (AD, frontotemporal lobe dementia, and Lewy body disease), whereas 45 metabolites detected in serum could differentiate at least one pair of these dementia groups [59]. More recently, the same methodology was employed to discriminate between AD and idiopathic normal pressure hydrocephalus patients by analyzing CSF [60].

## 6. Alzheimer’s Disease and Other RPLC-MS Based Platforms to Explore Central Metabolites

As previously described, there are different strategies to reduce the polarity of hydrophilic metabolites for enabling their analysis by means of RPLC-MS. For instance, Kaddurah-Daouk et al. published various metabolomics studies based on ion pairing and subsequent electrochemical array detection for studying the involvement of redox-active CSF metabolites on AD and MCI [61,62]. Alternatively, dansylation has also been employed as a derivatization procedure for enhancing the resolving power of RPLC for the analysis of AD-related central metabolites in saliva [63] and urine [64] samples. In this regard, Takayama et al. developed an alternative approach for chiral metabolomics by using optically active derivatization reagents, which enabled determining chiral amines and carboxyls in brain samples as biomarker candidates for AD diagnosis [65]. To conclude, it should also be noted that various manufacturers have developed novel RPLC stationary phases with an improved retention of hydrophilic compounds, which have been successfully employed for analyzing cationic metabolites in CSF [66] and purine metabolites in brain [67] of AD patients.

## 7. Overview on the Involvement of Central Metabolic Pathways in Alzheimer’s Disease

**Figure 1:**
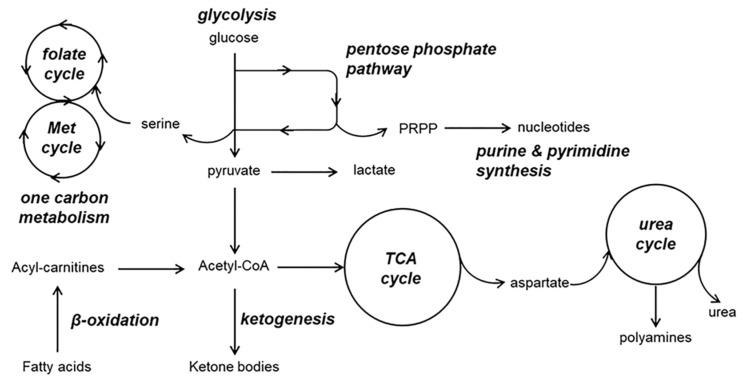
Overview of the central metabolic pathways altered in Alzheimer’s disease.

As shown in Table 1, hydrophilic-oriented MS-based metabolomics has demonstrated significant alterations in the levels of numerous polar metabolites in various biological matrices (e.g., serum/plasma, CSF, brain) and, consequently, the involvement of central metabolic pathways in the pathogenesis of AD (Figure 1). One of the most consistent findings across the metabolomics studies that have been reviewed here is the impairment of the energy-related metabolism. Altered glucose levels have been repeatedly reported in both the central nervous system and the peripheral system, suggesting an abnormal metabolic rate of carbohydrates, which are the main energy source in the brain. In turn, this was normally accompanied by perturbations in other metabolites participating in the glycolysis, the pentose phosphate pathway, ketogenesis and gluconeogenesis, the tricarboxylic (TCA) cycle, β-oxidation of fatty acids, and others (Table 1), thus evidencing profound disturbances affecting the entire energy metabolic system. Amino acids are involved in multiple central metabolic pathways, many of which have also been associated to the onset and progression of AD. It is noteworthy that failures in the homeostasis of aromatic amino acids and the synthesis of neurotransmitters (e.g., dopamine from tyrosine, serotonin from tryptophan) have been consistently reported in the literature. In this respect, growing evidence supports a role of branched chain amino acids in AD pathogenesis because these can compete with aromatic amino acids for entry into the brain but also due to their crucial involvement in the modulation of insulin resistance and energy metabolism. Moreover, various amino acids (e.g., arginine, glutamate/glutamine system) are closely linked to the nitrogen metabolism through the urea cycle and the polyamine system, which may also play a major role in brain health (e.g., hyperammonemia-induced neurotoxicity). Oxidative stress is another pivotal hallmark of AD with great impact on the metabolome, inducing the reduction of cerebral and circulating levels of numerous antioxidant metabolites (e.g., glutathione) and the accumulation of by-products derived from the oxidative damage to nucleic acids, proteins, and lipids. In this vein, various authors have reported important deregulations in the metabolism of purines and pyrimidines, which could be allocated not only to oxidative/nitrosative damage to nucleic acids but also to energy metabolism failures and impaired cellular signaling. In the crosstalk of many of the metabolic processes described above, including the homeostasis of amino acids, the synthesis of purines and redox defense, the one-carbon metabolism is also considered an essential piece of the AD pathology puzzle, in which hyperhomocysteinemia is one of the most important risk factors for cognitive decline and dementia. Altogether, the application of complementary metabolomics platforms for characterizing the polar metabolome stands out as a powerful strategy to decipher the molecular events behind the multifactorial pathogenesis and progression of AD. 

## 8. Conclusions

Hydrophilic metabolites play an essential role in the central and primary pathways of the human metabolism (e.g., metabolism of carbohydrates, amino acids, and fatty acids). To approach this pivotal portion of the human metabolome, the coupling of mass spectrometry with gas chromatography (GC-MS), hydrophilic interaction liquid chromatography (HILIC-MS), and capillary electrophoresis (CE-MS) are currently the analytical techniques most commonly employed, together with direct mass spectrometry (DMS) analysis for comprehensive and high-throughput metabolomics. DMS can be regarded as a first-pass screening tool for rapid and simple metabolomics fingerprinting, but it usually requires being complemented with other hyphenated MS approaches to overcome its inherent analytical limitations (e.g., the impossibility of resolving isomeric metabolites, ion suppression) in order to get a deeper insight into the polar metabolome. In this respect, the coupling GC-MS is the most commonly used platform for profiling low molecular weight hydrophilic metabolites because of its sensitivity and reproducibility, despite the need for a derivatization step prior to analysis. As an alternative, HILIC and CE provide orthogonal separation performance for polar and ionic metabolites, but significant technical developments are still needed for increasing their robustness and high-throughput capacity. The application of these metabolomics platforms has demonstrated the great impact of the onset and progression of AD on central metabolites and associated metabolic pathways, encompassing disturbances in the energy-related metabolism (e.g., glycolysis, TCA cycle), nitrogen metabolism (e.g., urea cycle, polyamine metabolism), fatty acid metabolism (e.g., β-oxidation, eicosanoids), neurotransmission (e.g., serotonergic, dopaminergic), homeostasis of amino acids and nucleotides, and some others. These findings are of utmost importance for comprehensively understanding the pathogenesis of this neurodegenerative disorder with the aim of developing possible therapeutic and preventive approaches and for discovering potential diagnostic targets. However, it should be noted that unsatisfactory validation studies have been repeatedly reported in AD metabolomics [70,71,72]. These inconsistencies can in part arise from analytical issues related to the lack of proper standardization in metabolomics research but also to the enormous intra- and inter-individual variability of the human metabolome. Therefore, many authors have emphasized in recent years the great potential of metabolomics to comprehensively investigate biological pathways and the etiopathology of diseases but also the extreme difficulty of using metabolites as robust biomarkers for diagnosis/prognosis in the clinical practice [73].

## Figures and Tables

**Table 1:** Summary of hydrophilic-oriented metabolomics studies on Alzheimer’s disease.

Study Population	Analytical Platform	Biological Sample	Key Results (Altered Pathways)	Ref.
AD (N = 22)/healthy controls (N = 18)	DMS	serum	Energy metabolism (glucose, carnitine, creatine), fatty acid metabolism (free fatty acids, eicosanoids), neurotransmission (dopamine), phospholipid homeostasis	[18]
AD (N = 22)/healthy controls (N = 18)	DMS	serum	Phospholipid homeostasis	[19]
AD (N = 22)/healthy controls (N = 18)	DMS	serum	Nitrogen metabolism (guanidine, arginine, putrescine), fatty acid metabolism (eicosanoids), neurotransmission (kynurenine), phospholipid homeostasis	[20]
AD (N = 19)/healthy controls (N = 17)	DMS + RPLC-MS	serum	Phospholipid homeostasis	[21]
AD (N = 30)/healthy controls (N = 30)	DMS (APPI)	serum	Energy metabolism (creatine, malic acid), fatty acid metabolism (free fatty acids, fatty acid amides), neurotransmission (dopamine, serotonin, picolinic acid), phospholipid and sphingolipid homeostasis	[22]
APP × PS1 (N = 30)/WT (N = 30)	DMS (ESI+APPI)	serum	Energy metabolism (glucose, carnitine, creatine), fatty acid metabolism (free fatty acids, eicosanoids), nitrogen metabolism (urea), amino acid metabolism, lipid homeostasis	[23]
APP × PS1 (N = 10)/WT (N = 10)	DMS	urine	Unidentified discriminant signals	[24]
APP × PS1 (N = 30)/WT (N =30)	DMS	hippocampus, cortex, cerebellum, olfactory bulb	Energy metabolism (pyruvic acid), fatty acid metabolism (free fatty acids, acyl-carnitines, eicosanoids), nucleotide metabolism, nitrogen metabolism (urea, N-acetylspermidine), amino acid metabolism, neurotransmission (dopamine), phospholipid homeostasis	[25]
APP × PS1 (N = 30)/WT (N = 30)	DMS	liver, kidney, spleen, thymus	Energy metabolism (glycolysis, TCA, creatine), fatty acid metabolism (free fatty acids, acyl-carnitines, eicosanoids), nucleotide metabolism, nitrogen metabolism (urea, polyamines), amino acid metabolism, lipid homeostasis	[26]
APP × PS1 × IL4-KO (N = 7)/APP × PS1 (N = 7)/WT (N = 7)	DMS	serum	Fatty acid metabolism (eicosanoids), nitrogen metabolism (urea, citrulline), amino acid metabolism, neurotransmission (dopamine, histamine)	[27]
CRND8 (N = 6)/WT (N = 6)	DMS	hippocampus	Energy metabolism (glucose), fatty acid metabolism (eicosanoids, β-oxidation)	[28]
CRND8 (N = 6)/WT (N = 6)	DMS	cerebellum	Fatty acid metabolism (eicosanoids), amino acid metabolism, nucleotide metabolism (purines)	[29]
AD (N = 9)/healthy controls (N = 9)	GC-MS	hippocampus, entorhinal cortex, middle-temporal gyrus, sensory cortex, motor cortex, cingulate gyrus, cerebellum	Energy metabolism (glycolysis, pentose phosphate, TCA), nucleotide metabolism, nitrogen metabolism (urea), amino acid metabolism	[30]
SAMP8 (N = 5, 2 months; N = 6, 7 months; N = 7, 12 months)	GC-MS	hippocampus	Energy metabolism (TCA, lactic acid), nitrogen metabolism (urea), amino acid metabolism, lipid homeostasis	[31]
APP × PS1 (N = 12)/WT (N = 11)	GC-MS	hippocampus	Energy metabolism (ketone bodies), amino acid metabolism, sphingolipid homeostasis	[32]
TASTPM (N = 16)/WT (N = 5)	GC-MS	whole brain, plasma	Energy metabolism (glycolysis, pentose phosphate), amino acid metabolism, steroid homeostasis	[33]
AD (N = 23)/healthy controls (N = 21)	GC-MS	serum	Energy metabolism (glucose, TCA, lactic acid), fatty acid metabolism (free fatty acids), nucleotide metabolism, nitrogen metabolism (urea, ornithine), amino acid metabolism	[34]
AD (N = 24)/MCI (N = 16)/PD (N = 22)/healthy controls (N = 8)	GC-MS	exhaled breath	Phenol (PD)	[35]
APP_Tg2576_ (N = 15)/CRND8 (N = 9)/ APP_V717I_ (N = 10)/WT (N = 17 + 9 + 12)	GC-MS	urine	Urinary odorants	[36]
AD (N = 47)/MCI (N = 143)/healthy controls (N = 46)	GC-MS + RPLC-MS	serum	Baseline: lipid homeostasis (phospholipids, sphingolipids, sterols) Progression: energy metabolism (2,4-dihydroxybutanoic acid)	[37]
AD (N = 57)/MCI (N = 58)/healthy controls (N = 57)	GC-MS + RPLC-MS	plasma	Fatty acid metabolism (free fatty acids), energy metabolism (glycolysis, TCA), one-carbon metabolism, amino acid metabolism, nucleotide metabolism	[38]
DS-AD (N = 78)/DS-control (N = 68)	GC-MS + RPLC-MS	plasma	Energy metabolism (anaerobic respiration)	[39]
APP_Tg2576_ (N = 3)/PS1 (N = 3)/APP × PS1 (N = 6)/WT (N = 6)	GC-MS + RPLC-MS	hippocampus	Energy metabolism (glycolysis, TCA), nucleotide metabolism, amino acid metabolism, neurotransmission	[40]
AD (N = 79)/healthy controls (N = 51)	GC-MS + RPLC-MS	CSF	Neurotransmission (dopamine, noradrenaline, MHPG), cortisol, uridine	[41]
AD (N = 40)/healthy controls (N = 38)	GC-MS + RPLC-MS	CSF	Two unidentified discriminant signals	[42]
APP × PS1 (N = 30)/WT (N = 30)	GC-MS + RPLC-MS	serum	Energy metabolism (glycolysis, TCA), fatty acid metabolism (free fatty acids, fatty acid amides, acyl-carnitines, eicosanoids), nitrogen metabolism (urea, citrulline), nucleotide metabolism, amino acid metabolism, neurotransmission (serotonin), homeostasis of cholesterol, phospholipids and sphingolipids	[43]
APP × PS1 (N = 30)/WT (N = 30)	GC-MS + RPLC-MS	hippocampus, cortex, striatum, cerebellum, olfactory bulb	Energy metabolism (glycolysis, TCA), nitrogen metabolism (urea), amino acid metabolism, neurotransmission (dopamine), phospholipid and sphingolipid homeostasis	[44]
APP × PS1 (N = 30)/WT (N = 30)	GC-MS + RPLC-MS	liver, kidney	Energy metabolism (glycolysis, TCA), fatty acid metabolism (free fatty acids, acyl-carnitines), nitrogen metabolism (urea, spermidine), amino acid metabolism, homeostasis of cholesterol, phospholipids and sphingolipids	[45]
APP × PS1 (N = 30)/WT (N = 30)	GC-MS + RPLC-MS	spleen, thymus	Energy metabolism (glycolysis, TCA), fatty acid metabolism (free fatty acids, acyl-carnitines), nitrogen metabolism (urea, putrescine), nucleotide metabolism, amino acid metabolism, homeostasis of cholesterol, phospholipids and sphingolipids	[46]
AD (N = 15)/healthy controls (N = 15)	HILIC-MS	neocortex	76 unidentified discriminant signals	[47]
AD (N =20)/healthy controls (N = 20)	HILIC-MS	plasma	54 unidentified discriminant signals	[48]
MCI_AD (N = 19)/MCI (N = 16)/healthy controls (N = 37)	HILIC-MS	plasma	Polyamine metabolism, L-arginine metabolism	[49]
CRND8 (N = 18/12, 12/18 weeks)/WT (N = 12/12, 12/18 weeks)	HILIC-MS	urine	Aromatic amino acid metabolism, nucleotide metabolism, ascorbate metabolism	[50]
AD (N = 15)/MCI (N = 15)/healthy controls (N = 15)	HILIC-MS + RPLC-MS	plasma, CSF	Energy metabolism (glycolysis, TCA), fatty acid metabolism, amino acid metabolism, neurotransmission, lipid homeostasis	[51]
AD (N = 21)/MCI_AD (N = 12)/MCI_stable (N = 21)/healthy controls (N = 21)	HILIC-MS + RPLC-MS	CSF	Nucleotide metabolism, amino acid metabolism, neurotransmission	[52]
AD (N = 9)/healthy controls (N = 9)	HILIC-MS + RPLC-MS	superior temporal cortex	Amino acid metabolism, neurotransmission	[53]
AD (N = 21)/healthy controls (N = 19)	HILIC-MS + RPLC-MS	frontal cortex	Amino acid metabolism, purine metabolism, pantothenate and CoA biosynthesis, phospholipid homeostasis	[54]
AD (N = 30)/MCI (N = 30)/healthy controls (N = 30)	HILIC-MS + RPLC-MS	plasma	Sphingolipid metabolism	[55]
AD (N = 23)/MCI_AD (N = 9)/MCI_stable (N = 22)/SCI (N = 19)	CE-MS	CSF	Amino acid metabolism, fatty acid metabolism, one-carbon metabolism	[56]
AD (N = 42)/MCI (N = 14)/healthy controls (N = 37)	CE-MS	serum	Amino acid metabolism, fatty acid metabolism, one-carbon metabolism	[57]
AD (N = 17)/asymptomatic AD (N = 13)/healthy controls (N = 13)	CE-MS	inferior temporal gyrus, middle frontal gyrus, cerebellum	Nitrogen metabolism (urea, polyamines), one-carbon metabolism, neurotransmission	[58]
AD (N = 3)/FTLD (N = 4)/LBD (N = 3)/healthy controls (N = 9)	CE-MS	serum, saliva	Energy metabolism, amino acid metabolism	[59]
AD (N = 81)/iNPH (N = 57)	CE-MS	CSF	Energy metabolism, amino acid metabolism	[60]
AD (N = 15)/healthy controls (N = 15)	RPLC-MS (ion pairing)	CSF	Neurotransmission, nucleotide metabolism, antioxidant defense	[61]
AD (N= 40)/MCI (N = 36)/healthy controls (N = 38)	RPLC-MS (ion pairing)	CSF	Neurotransmission, nucleotide metabolism, antioxidant defense	[62]
MCI (N = 20)/healthy controls (N = 20)	RPLC-MS (derivatization)	saliva	Taurine	[63]
CRND8 (N = 12)/WT (N = 12)	RPLC-MS (derivatization)	urine	Taurine, amino acid metabolism	[64]
AD_younger (N = 4)/AD_older (N = 4)/healthy controls (N = 3)	RPLC-MS (derivatization)	frontal lobe	L-phenylalanine, L-lactate	[65]
AD (N = 17)/healthy controls (N = 17)	RPLC-MS (improved retention for polar metabolites)	CSF	53 unidentified discriminant signals	[66]
AD I-II (N = 7)/AD III-IV (N = 4)/AD V-VI (N = 5)/healthy controls (N = 4)	RPLC-MS (improved retention for polar metabolites)	entorhinal cortex	Nucleotide metabolism	[67]

## References

[1] Blennow K., de Leon M.J., Zetterberg H. (2006). Alzheimer’s disease. Lancet.

[2] Maccioni R.B., Muñoz J.P., Barbeito L. (2001). The molecular bases of Alzheimer’s disease and other neurodegenerative disorders. Arch. Med. Res..

[3] González-Domínguez R., García-Barrera T., Gómez-Ariza J.L. (2014). Characterization of metal profiles in serum during the progression of Alzheimer’s disease. Metallomics.

[4] González-Domínguez R., Sayago A., Fernández-Recamales Á. (2017). Metabolomics in Alzheimer’s disease: The need of complementary analytical platforms for the identification of biomarkers to unravel the underlying pathology. J. Chromatogr. B.

[5] Wilkins J.M., Trushina E. (2017). Application of metabolomics in Alzheimer’s disease. Front. Neurol..

[6] Emwas A.H.M., Salek R.M., Griffin J.L., Merzaban J. (2013). NMR-based metabolomics in human disease diagnosis: Applications, limitations, and recommendations. Metabolomics.

[7] González-Domínguez R., Jáuregui O., Queipo-Ortuño M.I., Andrés-Lacueva C. (2020). Characterization of the human exposome by a comprehensive and quantitative large-scale multianalyte metabolomics platform. Anal. Chem..

[8] Wei F., Lamichhane S., Orešič M., Hyötyläinen T. (2019). Lipidomes in health and disease: Analytical strategies and considerations. Trends Anal. Chem..

[9] Zeng Y., Luo L., Hou W., Lu B., Gong J., Chen J., Zhang X., Han B., Xie Z., Liao Q. (2017). Targeted metabolomics analysis of aromatic amino acids and their gut microbiota-host cometabolites in rat serum and urine by liquid chromatography coupled with tandem mass spectrometry. J. Sep. Sci..

[10] González-Domínguez R., Urpi-Sarda M., Jáuregui O., Needs P.W., Kroon P.A., Andrés-Lacueva C. (2020). Quantitative Dietary Fingerprinting (QDF)-A novel tool for comprehensive dietary assessment based on urinary nutrimetabolomics. J. Agric. Food Chem..

[11] González-Domínguez R., Jáuregui O., Mena P., Hanhineva K., Tinahones F.J., Angelino D., Andrés-Lacueva C. (2020). Quantifying the human diet in the crosstalk between nutrition and health by multi-targeted metabolomics of food and microbiota-derived metabolites. Int. J. Obes..

[12] González-Domínguez R., Sayago A., Fernández-Recamales Á. (2017). Direct infusion mass spectrometry for metabolomic phenotyping of diseases. Bioanalysis.

[13] Beale D.J., Pinu F.R., Kouremenos K.A., Poojary M.M., Narayana V.K., Boughton B.A., Kanojia K., Dayalan S., Jones O.A.H., Dias D.A. (2018). Review of recent developments in GC-MS approaches to metabolomics-based research. Metabolomics.

[14] Tang D.Q., Zou L., Yin X.X., Ong C.N. (2016). HILIC-MS for metabolomics: An attractive and complementary approach to RPLC-MS. Mass Spectrom. Rev..

[15] Barbas C., Moraes E.P., Villaseñor A. (2011). Capillary electrophoresis as a metabolomics tool for non-targeted fingerprinting of biological samples. J. Pharm. Biomed. Anal..

[16] Chen K., Baluya D., Tosun M., Li F., Maletic-Savatic M. (2019). Imaging Mass Spectrometry: A New Tool to Assess Molecular Underpinnings of Neurodegeneration. Metabolites.

[17] Gonzalez-Dominguez A., Duran-Guerrero E., Fernandez-Recamales A., Lechuga-Sancho A.M., Sayago A., Schwarz M., Segundo C., Gonzalez-Dominguez R. (2017). An overview on the importance of combining complementary analytical platforms in metabolomic research. Curr. Top. Med. Chem..

[18] González-Domínguez R., García-Barrera T., Gómez-Ariza J.L. (2014). Using direct infusion mass spectrometry for serum metabolomics in Alzheimer’s disease. Anal. Bioanal. Chem..

[19] González-Domínguez R., García-Barrera T., Gómez-Ariza J.L. (2012). Metabolomic approach to Alzheimer’s disease diagnosis based on mass spectrometry. Chem. Pap..

[20] González-Domínguez R., García-Barrera T., Gómez-Ariza J.L. (2014). Metabolomic study of lipids in serum for biomarker discovery in Alzheimer’s disease using direct infusion mass spectrometry. J. Pharm. Biomed. Anal..

[21] González-Domínguez R., García-Barrera T., Gómez-Ariza J.L. (2014). Combination of metabolomic and phospholipid-profiling approaches for the study of Alzheimer’s disease. J. Proteom..

[22] González-Domínguez R., García-Barrera T., Gómez-Ariza J.L. (2015). Application of a novel metabolomic approach based on atmospheric pressure photoionization mass spectrometry using flow injection analysis for the study of Alzheimer’s disease. Talanta.

[23] González-Domínguez R., García-Barrera T., Vitorica J., Gómez-Ariza J.L. (2015). Application of metabolomics based on direct mass spectrometry analysis for the elucidation of altered metabolic pathways in serum from the APP/PS1 transgenic model of Alzheimer’s disease. J. Pharm. Biomed. Anal..

[24] González-Domínguez R., Castilla-Quintero R., García-Barrera T., Gómez-Ariza J.L. (2014). Development of a metabolomic approach based on urine samples and direct infusion mass spectrometry. Anal. Biochem..

[25] González-Domínguez R., García-Barrera T., Vitorica J., Gómez-Ariza J.L. (2015). Metabolomic screening of regional brain alterations in the APP/PS1 transgenic model of Alzheimer’s disease by direct infusion mass spectrometry. J. Pharm. Biomed. Anal..

[26] González-Domínguez R., García-Barrera T., Vitorica J., Gómez-Ariza J.L. (2015). High throughput multiorgan metabolomics in the APP/PS1 mouse model of Alzheimer’s disease. Electrophoresis.

[27] González-Domínguez R., García-Barrera T., Vitorica J., Gómez-Ariza J.L. (2015). Metabolomic research on the role of interleukin-4 in Alzheimer’s disease. Metabolomics.

[28] Lin S., Liu H., Kanawati B., Liu L., Dong J., Li M., Huang J., Schmitt-Kopplin P., Cai Z. (2013). Hippocampal metabolomics using ultrahigh-resolution mass spectrometry reveals neuroinflammation from Alzheimer’s disease in CRND8 mice. Anal. Bioanal. Chem..

[29] Lin S., Kanawati B., Liu L., Witting M., Li M., Huang J., Schmitt-Kopplin P., Cai Z. (2014). Ultrahigh resolution mass spectrometry-based metabolic characterization reveals cerebellum as a disturbed region in two animal models. Talanta.

[30] Xu J., Begley P., Church S.J., Patassini S., Hollywood K.A., Jüllig M., Curtis M.A., Waldvogel H.J., Faull R.L., Unwin R.D. (2016). Graded perturbations of metabolism in multiple regions of human brain in Alzheimer’s disease: Snapshot of a pervasive metabolic disorder. Biochim. Biophys. Acta.

[31] Wang H., Lian K., Han B., Wang Y., Kuo S.H., Geng Y., Qiang J., Sun M., Wang M. (2014). Age-related alterations in the metabolic profile in the hippocampus of the senescence-accelerated mouse prone 8: A spontaneous Alzheimer’s disease mouse model. J. Alzheimers Dis..

[32] Han B., Wang J.H., Geng Y., Shen L., Wang H.L., Wang Y.Y., Wang M.W. (2017). Chronic stress contributes to cognitive dysfunction and hippocampal metabolic abnormalities in APP/PS1 mice. Cell Physiol. Biochem..

[33] Hu Z.P., Browne E.R., Liu T., Angel T.E., Ho P.C., Chan E.C.Y. (2012). Metabonomic profiling of TASTPM transgenic Alzheimer’s disease mouse model. J. Proteome Res..

[34] González-Domínguez R., García-Barrera T., Gómez-Ariza J.L. (2015). Metabolite profiling for the identification of altered metabolic pathways in Alzheimer’s disease. J. Pharm. Biomed. Anal..

[35] Ko P.W., Kang K., Yu J.B., Huh J.S., Lee H.W., Lim J.O. (2014). Breath gas analysis for a potential diagnostic method of neurodegenerative diseases. Sens. Lett..

[36] Kimball B.A., Wilson D.A., Wesson D.W. (2016). Alterations of the volatile metabolome in mouse models of Alzheimer’s disease. Sci. Rep..

[37] Orešič M., Hyötyläinen T., Herukka S.K., Sysi-Aho M., Mattila I., Seppänan-Laakso T., Julkunen V., Gopalacharyulu P.V., Hallikainen M., Koikkalainen J. (2011). Metabolome in progression to Alzheimer’s disease. Transl. Psychiatry.

[38] Wang G., Zhou Y., Huang F.J., Tang H.D., Xu X.H., Liu J.J., Wang Y., Deng Y.L., Ren R.J., Xu W. (2014). Plasma metabolite profiles of Alzheimer’s disease and mild cognitive impairment. J. Proteome Res..

[39] Gross T.J., Doran E., Cheema A.K., Head E., Lott I.T., Mapstone M. (2019). Plasma metabolites related to cellular energy metabolism are altered in adults with Down syndrome and Alzheimer’s disease. Dev. Neurobiol..

[40] Trushina E., Nemutlu E., Zhang S., Christensen T., Camp J., Mesa J., Siddiqui A., Tamura Y., Sesaki H., Wengenack T.M. (2012). Defects in mitochondrial dynamics and metabolomic signatures of evolving energetic stress in mouse models of familial Alzheimer’s disease. PLoS ONE.

[41] Czech C., Berndt P., Busch K., Schmitz O., Wiemer J., Most V., Hampel H., Kastler J., Senn H. (2012). Metabolite profiling of Alzheimer’s disease cerebrospinal fluid. PLoS ONE.

[42] Motsinger-Reif A.A., Zhu H., Kling M.A., Matson W., Sharma S., Fiehn O., Reif D.M., Appleby D.H., Doraiswamy P.M., Trojanowski J.Q. (2013). Comparing metabolomic and pathologic biomarkers alone and in combination for discriminating Alzheimer’s disease from normal cognitive aging. Acta Neuropathol. Commun..

[43] González-Domínguez R., García-Barrera T., Vitorica J., Gómez-Ariza J.L. (2015). Deciphering metabolic abnormalities associated with Alzheimer’s disease in the APP/PS1 mouse model using integrated metabolomic approaches. Biochimie.

[44] González-Domínguez R., García-Barrera T., Vitorica J., Gómez-Ariza J.L. (2014). Region-specific metabolic alterations in the brain of the APP/PS1 transgenic mice of Alzheimer’s disease. Biochim. Biophys. Acta.

[45] González-Domínguez R., García-Barrera T., Vitorica J., Gómez-Ariza J.L. (2015). Metabolomic investigation of systemic manifestations associated with Alzheimer’s disease in the APP/PS1 transgenic mouse model. Mol. BioSyst..

[46] González-Domínguez R., García-Barrera T., Vitorica J., Gómez-Ariza J.L. (2015). Metabolomics reveals significant impairments in the immune system of the APP/PS1 transgenic mice of Alzheimer’s disease. Electrophoresis.

[47] Graham S.F., Chevallier O.P., Roberts D., Holscher C., Elliot C.T., Green B.D. (2013). Investigation of the human brain metabolome to identify potential markers for early diagnosis and therapeutic targets of Alzheimer’s disease. Anal. Chem..

[48] Inoue K., Tsuchiya H., Takayama T., Akatsu H., Hashizume Y., Yamamoto T., Matsukawa N., Toyo’oka T. (2015). Blood-based diagnosis of Alzheimer’s disease using fingerprinting metabolomics based on hydrophilic interaction liquid chromatography with mass spectrometry and multivariate statistical analysis. J. Chromatogr. B.

[49] Graham S.F., Chevallier O.P., Elliot C.T., Hölscher C., Johnston J., McGuinness B., Kehoe P.G., Passmore A.P., Green B.D. (2015). Untargeted metabolomic analysis of human plasma indicates differentially affected polyamine and L-arginine metabolism in mild cognitive impairment subjects converting to Alzheimer’s disease. PLoS ONE.

[50] Tang Z., Liu L., Li Y., Dong J., Li M., Huang J., Lin S., Cai Z. (2016). Urinary metabolomics reveals alterations of aromatic amino acid metabolism of Alzheimer’s disease in the transgenic CRND8 mice. Curr. Alzheimer Res..

[51] Trushina E., Dutta T., Persson X.M., Mielke M.M., Petersen R.C. (2013). Identification of altered metabolic pathways in plasma and CSF in mild cognitive impairment and Alzheimer’s disease using metabolomics. PLoS ONE.

[52] Ibañez C., Simo C., Barupal D.K., Fiehn O., Kivipelto M., Cedazo-Mínguez A., Cifuentes A. (2013). A new metabolomic workflow for early detection of Alzheimer’s disease. J. Chromatogr. A.

[53] Kim Y.H., Shim H.S., Kim K.H., Lee J., Chung B.C., Kowall N.W., Ryu H., Lee J. (2019). Metabolomic analysis identifies alterations of amino acid metabolome signatures in the postmortem brain of Alzheimer’s disease. Exp. Neurobiol..

[54] Paglia G., Stocchero M., Cacciatore S., Lai S., Angel P., Alam M.T., Keller M., Ralser M., Astarita G. (2016). Unbiased metabolomic investigation of Alzheimer’s disease brain points to dysregulation of mitochondrial aspartate metabolism. J. Proteome Res..

[55] Armirotti A., Basit A., Realini N., Caltagirone C., Bossù P., Spalletta G., Piomelli D. (2014). Sample preparation and orthogonal chromatography for broad polarity range plasma metabolomics: Application to human subjects with neurodegenerative dementia. Anal. Biochem..

[56] Ibáñez C., Simó C., Martín-Álvarez P.J., Kivipelto M., Winblad B., Cedazo-Mínguez A., Cifuentes A. (2012). Toward a predictive model of Alzheimer’s disease progression using capillary electrophoresis–mass spectrometry metabolomics. Anal. Chem..

[57] González-Domínguez R., García A., García-Barrera T., Barbas C., Gómez-Ariza J.L. (2014). Metabolomic profiling of serum in the progression of Alzheimer’s disease by capillary electrophoresis-mass spectrometry. Electrophoresis.

[58] Mahajan U.V., Varma V.R., Griswold M.E., Blackshear C.T., An Y., Oommen A.M., Varma S., Troncoso J.C., Pletnikova O., O’Brien R. (2020). Dysregulation of multiple metabolic networks related to brain transmethylation and polyamine pathways in Alzheimer disease: A targeted metabolomic and transcriptomic study. PLoS Med..

[59] Tsuruoka M., Hara J., Hirayama A., Sugimoto M., Soga T., Shankle W.R., Tomita M. (2013). Capillary electrophoresis-mass spectrometry-based metabolome analysis of serum and saliva from neurodegenerative dementia patients. Electrophoresis.

[60] Nagata Y., Hirayama A., Ikeda S., Shirahata A., Shoji F., Maruyama M., Kayano M., Bundo M., Hattori K., Yoshida S. (2018). Comparative analysis of cerebrospinal fluid metabolites in Alzheimer’s disease and idiopathic normal pressure hydrocephalus in a Japanese cohort. Biomark. Res..

[61] Kaddurah-Daouk R., Rozen S., Matson W., Han X., Hulette C.M., Burke J.R., Doraiswamy P.M., Welsh-Bohmer K.A. (2011). Metabolomic changes in autopsy-confirmed Alzheimer’s disease. Alzheimers Dement..

[62] Kaddurah-Daouk R., Zhu H., Sharma S., Bogdanov M., Rozen S.G., Matson W., Oki N.O., Motsinger-Reif A.A., Churchill E., Lei Z. (2013). Alterations in metabolic pathways and networks in Alzheimer’s disease. Transl. Psychiatry.

[63] Zheng J., Dixon R.A., Li L. (2012). Development of isotope labeling LC-MS for human salivary metabolomics and application to profiling metabolome changes associated with mild cognitive impairment. Anal. Chem..

[64] Peng J., Guo K., Xia J., Zhou J., Yang J., Westaway D., Wishart D.S., Li L. (2014). Development of isotope labeling liquid chromatography mass spectrometry for mouse urine metabolomics: Quantitative metabolomic study of transgenic mice related to Alzheimer’s disease. J. Proteome Res..

[65] Takayama T., Mochizuki T., Todoroki K., Min J.Z., Mizuno H., Inoue K., Akatsu H., Noge I., Toyo’oka T. (2015). A novel approach for LC-MS/MS-based chiral metabolomics fingerprinting and chiral metabolomics extraction using a pair of enantiomers of chiral derivatization reagents. Anal. Chim. Acta.

[66] Myint K.T., Aoshima K., Tanaka S., Nakamura T., Oda Y. (2009). Quantitative profiling of polar cationic metabolites in human cerebrospinal fluid by reversed-phase nanoliquid chromatography/mass spectrometry. Anal. Chem..

[67] Ansoleaga B., Jové M., Schlüter A., Garcia-Esparcia P., Moreno J., Pujol A., Pamplona R., Portero-Otín M., Ferrer I. (2015). Deregulation of purine metabolism in Alzheimer’s disease. Neurobiol. Aging.

[68] González-Domínguez R., Sayago A., Fernández-Recamales Á. (2018). High-throughput direct mass spectrometry-based metabolomics to characterize metabolite fingerprints associated with Alzheimer’s disease pathogenesis. Metabolites.

[69] Low D.Y., Lefèvre-Arbogast S., González-Domínguez R., Urpi-Sarda M., Micheau P., Petera M., Centeno D., Durand S., Pujos-Guillot E., Korosi A. (2019). Diet-related metabolites associated with cognitive decline revealed by untargeted metabolomics in a prospective cohort. Mol. Nutr. Food Res..

[70] Casanova R., Varma S., Simpson B., Min K., An Y., Saldana S., Riveros C., Moscato P., Griswold M., Sonntag D. (2016). Blood metabolite markers of preclinical Alzheimer’s disease in two longitudinally followed cohorts of older individuals. Alzheimers Dement..

[71] Li D., Misialek J.R., Boerwinkle E., Gottesman R.F., Sharrett A.R., Mosley T.H., Coresh J., Wruck L.M., Knopman D.S., Alonso A. (2017). Prospective associations of plasma phospholipids and mild cognitive impairment/dementia among African Americans in the ARIC Neurocognitive Study. Alzheimers Dement..

[72] Costa A.C., Joaquim H.P.G., Forlenza O., Talib L.L., Gattaz W.F. (2019). Plasma lipids metabolism in mild cognitive impairment and Alzheimer’s disease. World J. Biol. Psychiatry.

[73] Johnson C.H., Ivanisevic J., Siuzdak G. (2016). Metabolomics: Beyond biomarkers and towards mechanisms. Nat. Rev. Mol. Cell Biol..

